# An updated toolkit for exploring bacterial cell wall structure and dynamics

**DOI:** 10.12703/r/10-14

**Published:** 2021-02-10

**Authors:** Michael C Gilmore, Barbara Ritzl-Rinkenberger, Felipe Cava

**Affiliations:** 1 Laboratory for Molecular Infection Medicine Sweden (MIMS), Department of Molecular Biology, Umeå University, Umeå, Sweden

**Keywords:** peptidoglycan, ultra performance liquid chromatography, FDAA, AFM, Cryo-EM

## Abstract

The bacterial cell wall is made primarily from peptidoglycan, a complex biomolecule which forms a bag-like exoskeleton that envelops the cell. As it is unique to bacteria and typically essential for their growth and survival, it represents one of the most successful targets for antibiotics. Although peptidoglycan has been studied intensively for over 50 years, the past decade has seen major steps in our understanding of this molecule because of the advent of new analytical and imaging methods. Here, we outline the most recent developments in tools that have helped to elucidate peptidoglycan structure and dynamics.

## Introduction

In order to counteract their own internal turgor pressure, most bacteria synthesise a cell wall, which forms a bag-like exoskeleton that envelopes the cell. The cell wall is composed primarily of peptidoglycan (PG), a complex biopolymer which consists of glycan strands of alternating *N*-acetylmuramic acid (NAM) and *N*-acetylglucosamine (NAG) sugars cross-linked by short peptide chains, most commonly L-Ala-γ-D-Glu-*meso*-DAP-D-Ala-D-Ala (DAP, diaminopimelic acid) in Gram-negative bacteria or L-Ala-γ-D-Glu-L-Lys-D-Ala-D-Ala in Gram-positive bacteria^1^.

As it is a unique characteristic of bacteria and typically essential for their growth, the cell wall is the target for many of our most successful antibiotics, and its synthesis and regulation have been the subject of intensive research for over five decades. Its synthesis begins with the cytoplasmic assembly of a lipid-linked precursor, lipid II^2,3^, which is flipped to the external side of the cytoplasmic membrane to be polymerised and cross-linked into the mature PG mesh. This assembly is carried out by the penicillin-binding proteins (PBPs), so named as they are the target of β-lactam antibiotics^4^, and the more recently discovered penicillin-insensitive shape, elongation, division, and sporulation (SEDS) proteins^5^. Far from being static, the cell wall is constantly remodelled to facilitate growth and division and can be subject to modifications which confer protection against environmental threats and facilitate adaptation to the present niche^6^.

The most recent advances in understanding how PG is synthesised and regulated have been well reviewed elsewhere^7^. Here, we summarise the methods which have emerged in the last decade that have dramatically improved our understanding of cell wall structure and dynamics. In particular, we focus on methods for elucidating the chemical structure of PG as well as new tools for probing its synthesis and remodelling.

## Chemical methods

In the early 1950s, pioneering studies on the mechanism of penicillin by James T. Park revealed that penicillin treatment of *Staphylococcus aureus* led to the accumulation of a uridine-linked peptide (termed ‘Park’s nucleotide’), which was unusual in that it contained amino acids in both their L- and D-stereoisomers^8^. Coinciding studies showed that the cell wall of bacteria contained the same amino acids^9,10^, leading to the realisation in further work by Park and Jack L. Strominger that penicillin targeted cell wall biosynthesis and the dawn of the study of the bacterial cell wall^11^.

Methods for the purification of the PG sacculus and its enzymatic digestion for chemical analysis were established not long after its discovery^12^ and uncovered some variability in cell wall structure, particularly in Gram-positive bacteria in both the amino acid composition and presence of peptide bridges cross-linking the muropeptides (that is, disaccharide-peptide units)^13^. However, these methods were highly laborious and it was not until the development of high-performance liquid chromatography (HPLC) in the early 1980s that efficient muropeptide analysis and a better understanding of cell wall chemistry were made possible^14,15^, revealing the presence of a DAP-DAP or L,D cross-link in addition to the DAP-Ala or D,D cross-links which were previously known^14^. HPLC uses the relative affinity of solutes in a mobile phase for a stationary phase to separate them, which coupled with ultraviolet (UV)-spectrophotometric detection produces a chromatogram (the function of absorbance over time), allowing the identification of muropeptide species based on retention time. Integration of the peaks of the chromatogram provides a means by which to quantify the muropeptide species in terms of relative abundance, while the proportions of different muropeptides can provide information on the physical characteristics of the wall. High levels of dimeric and trimeric muropeptides correspond to more highly cross-linked and therefore stiffer PG, while a relative increase in anhydromuropeptides, which represent the glycan chain termini, corresponds to shorter glycan chains and more flexibility of the sacculus^16^.

While HPLC facilitated major steps in our understanding of PG, a new awareness of the metabolic diversity of bacteria brought a desire to understand how fine PG structure might adapt to environmental conditions. However, many of these conditions presented challenges for PG isolation, such as where infection, symbiosis or lab-unculturable bacteria mean that very low amounts of PG are available for analysis. As around 10^9^ to 10^10^ cells’ worth of PG are required for the detection of minor components in HPLC^16^, improved analytical techniques were needed. As a result, ultra-performance liquid chromatography (UPLC), which uses smaller columns with more compact stationary phases that enable the use of higher pressures, has superseded HPLC as it is able to achieve similar separation and resolution using an order of magnitude less sample, helping reveal the presence of very low amounts of PG in anammox Planctomycetes^17^.

**Figure 1.**
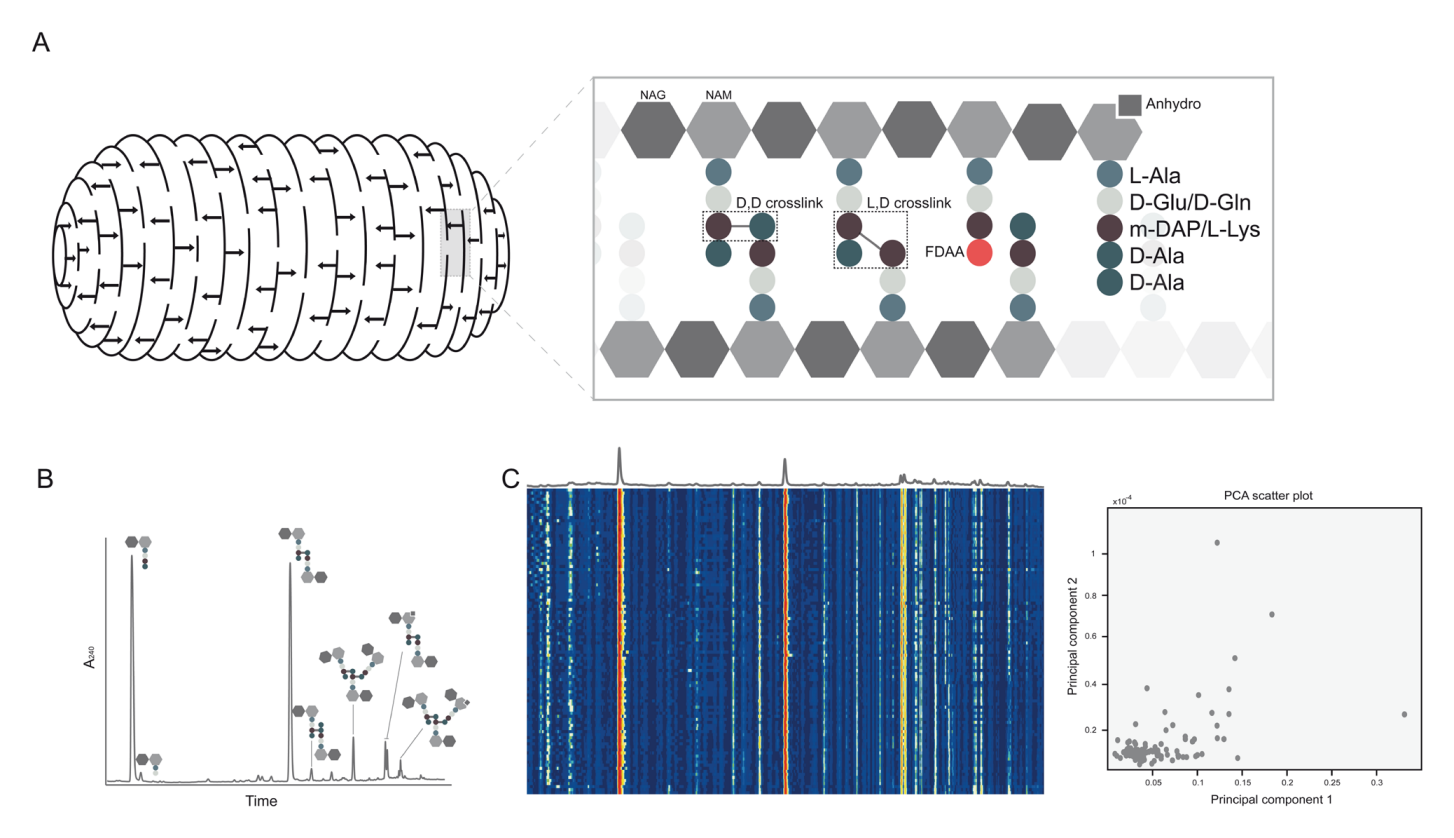
Overview of cell wall structure and high-throughput peptidoglycan (PG) analysis by ultra-performance liquid chromatography (UPLC). (**A**) Schematic diagram of PG sacculus showing glycan strands with arrows representing cross-links and chemical structure showing major features of PG, including DAP-Ala (D,D) and DAP-DAP (L,D) cross-links and an anhydromuropeptide. Also shown is the incorporation of a fluorescent D-amino acid (FDAA) in the fourth position. NAG, *N*-acetylglucosamine; NAM, *N*-acetylmuramic acid. (**B**) Representative UPLC chromatogram from PG of Gram-negative enteropathogen *Vibrio cholerae* with schematic muropeptide structures associated with each peak indicated. (**C**) Heatmap and principal component analysis (PCA) showing comparison of UPLC chromatograms from many transposon mutants of *V. cholerae*, highlighting how statistical tools can facilitate high-throughput PG analysis by indicating statistical outliers.

An important strength of UPLC over HPLC is its profoundly reduced run times; 2 hours taken previously by HPLC was lowered to potentially under 10 minutes. Robust protocols which allow the rapid isolation and analysis of muropeptides from bacteria by UPLC in under 24 hours have now been established^18,19^. The vastly reduced run times brought with UPLC had implications in the analysis of PG since they opened the gateway to high-throughput analysis of PG chemistry under many different conditions, or in many species, in relatively little time. However, the large amount of chromatographic data produced in these experiments necessitated the production of new tools to facilitate its comparison. The development of dedicated chemometric tools enabled many chromatograms to be aligned and compared and statistical outliers to be identified using principal component analysis or constraint randomised non-negative factor analysis^20,21^ (Figure 1). For example, a chemometric analysis of the chemical variability of PG in various Alphaproteobacteria revealed the presence of a new L,D-1,3 cross-link and mDAP amidation which can help protect Acetobacteria from Type VI secretion-associated PG endopeptidases and *Drosophila* innate immunity^20^. With these tools, it will be possible to analyse PG composition in a species under many different conditions, or in many different mutants, in a high-throughput manner, thereby revealing new factors in PG homeostasis (Figure 1).

The Gram-negative bacterium *Escherichia coli* has around 3.5 million muropeptides in its cell wall^22^. Given a limit of detection of 0.1 to 1% of the total muropeptide pool for UV-spectrophotometric detection, muropeptide species in orders of magnitude of 10^3^ to 10^4^ muropeptides per cell would not be detectable but could still be physiologically relevant. The coupling of UPLC with high-resolution mass spectrometry (MS) can allow the detection and identification of muropeptide species which are present in much smaller quantities, significantly lowering the previous detection limits achievable by UV and allowing rapid identification of muropeptide species without the need to collect peaks and analyse them separately by MS. Most modern instruments can automatically identify peaks by their mass and fragmentation spectra, an ability that lends itself to high-throughput screens for new PG chemistry. Tandem MS fragmentation analyses, where a target ion is broken into smaller fragment ions which then are separated, can aid in the identification of muropeptides, including those with known modifications. Since coupling UPLC with MS detection has much lower detection limits than UV alone, it can also be used for the analysis and quantification of intracellular muropeptides derived from *de novo* PG synthesis or PG recycling as well as the analysis of muropeptides that are released to the supernatant. This technique allows the facile and comprehensive analysis of released muropeptide species which can be important in the cell’s environment, such as in symbiont–host interactions or disease (reviewed in 23–27).

Nuclear magnetic resonance (NMR) has also been used as a tool to investigate PG chemistry in both elucidating the chemical structure of individual muropeptides^20^ and providing insights into the wider architecture of the PG polymer. The three-dimensional (3D) structure of the PG sacculus remains an outstanding problem in cell wall biology. An NMR study on the solution structure of a chemically synthesised two-muropeptide unit from the Mobashery lab showed that the glycan backbone formed a right-handed helix with threefold axial symmetry^28^, although that study was limited since the structure of dimeric muropeptides in solution may not correspond to that of cross-linked and load-bearing PG in the sacculus. As larger PG fragments are not typically soluble, solid-state NMR, which is not as widely available or sensitive as solution-state NMR, must instead be used. However, solid-state NMR can provide Ångstrom-resolution distance constraints which allow structure determination. The application of ^13^C spin diffusion centerband-only detection of exchange (CODEX) NMR to *S. aureus* PG *in situ* revealed a 5-Å distance between the pentaglycine bridge and a glycan chain of a neighbouring strand, evidence of a more tightly packed structure^29^. These data suggest that the glycan strands have fourfold axial symmetry, an organisation that permits a higher degree of cross-linking that corresponds more closely with observation. Subsequently, rotational-echo double-resonance (REDOR) solid-state NMR was applied to intact cells of *S. aureus* grown in the presence of D-[1-^13^C] alanine and L-[^15^N]-alanine with the addition of a ^19^F-labelled glycopeptide antibiotic, eremomycin 4-p-fluorophenylpeperazide, which binds specifically to PG. This allowed the distance between the L- and D-alanine moieties in neighbouring muropeptides to be measured as 4.4 Å, which coupled with a 4.8-Å distance between the terminal D-Ala of one muropeptide and bound glycopeptide of another lends further credence to this tightly packed arrangement^30,31^. A similar arrangement was observed in *Enteroccocus faecalis* under similar experimental conditions^32^.

## Imaging approaches

### Fluorescent labelling

In order to understand the spatiotemporal dynamics of PG synthesis and remodelling, molecular probes which would allow these processes to be visualised were needed. Historically, this was achieved using fluorescently labelled antibiotics^33^ or lectins^34^, which could be viewed using fluorescence microscopy, but these tools had significant drawbacks since they displayed low membrane permeability or toxicity towards the cell.

However, several reports have shown that muropeptides can be substituted with alternative, non-canonical amino acids in the fourth or fifth position by L,D transpeptidases or by PBPs and cytoplasmic ligases (that is, MurF, Ddl and VanA), respectively^35–37^, which opened the possibility for the incorporation of alternative, fluorescently labelled D-amino acids (FDAAs) into PG. Capitalising on this, the VanNieuwenhze and Brun laboratories designed and synthesised a panel of FDAAs, which consist of a D-amino acid backbone with the side chain replaced by a fluorophore^38,39^. As they are both biocompatible and highly specific, these probes have proven to be a powerful tool for labelling PG and as a result have entered widespread use.

Since FDAAs allow only transpeptidase-mediated PG labelling, probes that allowed the direct fluorescent labelling of lipid II were developed^40^. These took advantage of the conserved PG synthesis step of the addition of D-Ala-D-Ala (DA-DA) to the nascent tripeptide precursor by MurF, using a version of DA-DA with a functional handle that allows subsequent ‘click chemistry’ azide-alkyne cycloaddition of a fluorophore. These tools facilitated the resolution of the ‘Chlamydial anomaly’, in which the obligate intracellular pathogen *Chlamydia trachomatis* had no detectable PG despite possessing an almost complete set of PG biosynthetic genes, demonstrating that *C. trachomatis* in fact synthesises a ring-like PG structure. Other probes have used functionalised D-Ala derivatives which likewise can be used by the cell in PG precursor biosynthesis and subsequently conjugated to fluorophores or other detectable probes by bioorthogonal chemistry^41,42^. This principle has also been applied to the NAM sugar to produce a functionalised NAM which can be incorporated into the PG through the NAM recycling pathway and has an added advantage since, unlike D-Ala, it is not removed during PG remodelling, increasing the longevity of the probe, and meaning that it is also present in muropeptide species which lack D-Ala^43^.

A caveat of using FDAAs to label PG synthesis is that these reagents are continuously fluorescent and thus the method requires washing steps between pulses to remove free FDAAs. Recently, rotor-fluorogenic FDAAs (rfFDAAs), which fluoresce only in environments in which their rotation is restricted, have been synthesised^44^. As a result, rfFDAAs become fluorescent only on incorporation into the PG sacculus, allowing PG biosynthesis to be monitored in real time with a temporal resolution as high as the imaging method will allow while minimising the disturbance to the bacterial cells brought by repeated washing. Additionally, their ease of use in monitoring PG transpeptidation reactions lends itself to the development of high-throughput screens for inhibitors of these proteins.

### Microscopy techniques

Our knowledge of PG chemistry derived mainly from biochemical analyses, which profited largely from advances in chromatography methods. Muropeptide analysis by HPLC enabled us to know more about the general structure and biosynthesis of the cell wall components; however, many questions remained about the organisation, assembly and interaction of the cell wall constituents. Traditionally, these studies were conducted by electron microscopy (EM), which relies on a beam of accelerated electrons for illumination. Therefore, the production of a strong signal depends on the specimen itself. This would mean that a sample that does not contain electron-dense elements, such as PG, requires staining with heavy atoms for successful imaging. However, the treatments that are usually applied, such as staining and chemical fixation, can lead to damage of the sample and often can be misleading when it fails to maintain all structural macromolecules in their native structure^1,45^. Nevertheless, studies emerging from EM experiments indicated that isolated PG sacculi retain their shape^46^; this has proven to be useful in several different applications such as atomic force microscopy (AFM) and cryo-transmission EM (cryo-TEM) as it allowed the imaging of purified sacculi.

The invention of the AFM in the mid-1980s offered new opportunities to answer key questions in this field. In addition to improved resolution, AFM has several advantages compared with previous microscopy techniques. Sample preparation did not include any special treatment which might damage the murein, and it enabled 3D visualisation of the sacculi in a nearly native state. AFM proved to be a powerful tool to study different aspects of the cell wall properties and dynamics such as following the effects of drugs on the cell envelope^47^ and *in vivo* nano-imaging studies of the surface layer in *Corynebacterium glutamicum*^48^. Studies in this field are employing AFM to understand the architecture and organisation of PG, which is important to be able to provide models for bacterial growth and division, with a particular focus on the orientation of glycan chains. A significant part of this work has been carried out by the Foster group, who were able to show that PG structure is less ordered than previously thought^49^. In addition, the level of order changes when cell shape alterations are introduced to make *E. coli* cells spheroid, resulting in short and disordered glycan chains^49^.

Complementary to AFM and EM, cryo-TEM became an important tool over the years to directly visualise complex structures and provide information that is fundamental to understand them. Cryo-TEM enables preservation of higher-order structures and therefore facilitates new advances. Compared with conventional EM, samples are studied at cryogenic temperatures which do not interfere with the specimen’s state, enabling the preservation of native PG^50,51^. The development of an imaging technique, cryo-electron tomography (cryo-ET), made it possible to combine the images into a 3D projection^52^. Research in this field has made great progress answering questions that were in focus for decades but remained unresolved because of technical limitations, such as the molecular organisation of PG and how cells remodel their cell wall on the molecular level when they elongate and divide. With the help of cryo-ET, researchers were able to show that at least two Chlamydia species do possess a PG cell wall^53^ and revealed that the mycobacterial cell envelope consists of an inner cytoplasmic membrane as well as a symmetrical outer membrane^54,55^. Moreover, it provided information on the long-standing debate of the bacterial cell wall architecture. Findings of the Jensen lab supported a disorganised, layered model in Gram-negative bacteria, meaning that a single layer of glycans run circumferentially to the cell surface^56^. However, it left many questions open, especially when it comes to Gram-positive bacteria^57^. AFM studies conducted with *Bacillus subtilis* proposed a ‘coiled cable model’ in which bundles of glycan strands wrap around the cell^58^. However, questions about its organisation remained since cryo-EM results from Beeby *et al*. suggest that the structure of rod-shaped Gram-positive PG is not different from that of similarly shaped Gram-negative bacteria^59^. According to their results, multiple layers of PG stands run circumferentially along the long axis. Further AFM studies suggested that just as when bacteria undergo remodelling during different growth stages, there is a change in both cell wall architecture and thickness^60^. Although the authors could confirm the coiled cable model in stationary phase in *B. subtilis*, they described the side wall architecture more as a ‘ridge and groove’-like structure in mid-exponential phase. It is proposed that differences in the surface structure of the PG might vary as well between different bacterial strains. First high-resolution images of the bacterial cell wall gave further insight into the PG structure of Gram-positive bacteria (*B. subtilis* and *S. aureus*), revealing an unprecedented structure. With the help of high-resolution AFM, it has been shown that the external surface is composed of a landscape with large, deep pores. However, the internal surface seems to be much denser, and its organisation is also location-dependent. Moreover, the structural organisation also varies among bacterial species^61^. This is another example that shows the wide variety in PG architecture within different bacterial species. Research in this field could lead to a better understanding of the mechanical properties and the mode of action of drugs, especially antibiotics, on the cell wall of bacteria.

The lack of experimental techniques often creates a barrier in understanding complex processes and therefore it is crucial to provide insight into molecular and biophysical processes through computational modelling. These simulations have often been employed to understand various aspects of the cell wall properties^62^. Later approaches combined biophysical and biochemical data with computational modelling. With the help of so-called mesoscale modelling, researchers were able to simulate several hypotheses of molecular processes in their cellular environment^63^. This approach made it possible to simulate how Gram-negative bacteria can retain their shape and integrity during elongation^64^ as well as to explore how bacteria constrict their cell wall to divide^65^.

One of the biggest challenges in this field is trying to understand how bacterial cells grow, divide, remodel and recycle their PG, which has a fundamental impact not just in basic research but also in medicine. Owing to environmental changes or as part of their life cycle, cells constantly change their morphology and therefore often undergo changes in shape and size. These two features have been linked to various important behaviours. Cell shape has been shown to be important for predation, adhesion and motility^66^, whereas cell size has been proposed to have a role in host invasion. For example, some invasive pathogens such as *Streptococcus pneumoniae* and *Hemophilus influenzae* try to escape detection by the host by minimising their cell size^67,68^. Since cell shape and size are directly coupled to cell wall, a lot of effort has been put into image-based screens. Developments in automation and analysis software have made previously highly laborious screens possible. These observations in morphological changes led to the discovery of new gene functions, such as the discovery of RodZ, a cytoskeletal protein responsible for rod shape^69^. Since then, microscopy-based high-throughput screens were evolving fast, and a wide range of studies employ this technique to explore unknown cell shape determinants^70–72^. However, these studies are limited to single-time point analysis only. With the help of combined experimental and multivariate image analysis, profiling of morphological changes, such as antibiotic-induced changes, became available to follow^73^. To predict the responses to different perturbations and damages, analysis is often accompanied by mathematical or physical modelling, such as the physical model of *E. coli*^74^.

Another aspect of cell wall research focuses on the interplay between cell size determination and its effect on cellular physiology. Despite early studies, how cells regulate their shape, size and growth rate is still not entirely understood. As these questions are being unravelled, it is very important to ensure a controlled environment, preferably with a microfluidic flow cell to maintain a steady flux of nutrients. To achieve steady-state growth conditions and monitoring over a long time period, Si *et al*. introduced a multiplex turbidostat^75^. This enables high-throughput quantification of cells under diverse conditions. Another commonly applied microfluidics device is the mother machine. One could compare it to a comb where medium flows through the wide channel perpendicular to several narrow channels. Some cells get trapped in these narrow channels and serve as the ‘mother cell’ which proliferates up the narrow channel until reaching the top and being ‘washed away’ from the field of view by the medium. This way, cells can be imaged continuously for hundreds of generations^76^. Lately, these studies revealed that two evolutionarily distinct bacteria – *E. coli* and *B. subtilis* – share common principles to coordinate growth and cell cycle^77^.

As mentioned above, depending on the scope of the study, a fluorescence or a phase-contrast microscope can be coupled to the machine with automated image acquisition. For this purpose, software packages, such as MM3^78^, are freely available. However, automated microscopy experiments can generate immense data sets and can create a burden for image processing. To overcome this bottleneck, deep learning software has been introduced^79^.

As the changes in cell shape are closely coupled to the PG cell wall, many studies in recent years have tried to address the gaps in our knowledge of the spatiotemporal organisation of cell wall–related proteins and how cells maintain and regulate their structural properties. These events require precise coordination of multiprotein complexes. To understand the underlying mechanisms, sensitive methods needed to be developed. Cryo-EM played a great role in providing a better structural overview of known bacterial cytoskeletal elements as well as in the identification of some novel ones^80^. However, one pitfall of this technique is that it is not able to follow the dynamics of these proteins which would be vital for the understanding of these processes. Therefore, in addition to traditional fluorescence microscopy techniques, which often face limitations such as low resolution and sensitivity, a wide range of new microscopy techniques were developed. For bacterial cells, one of the commonly used microscopy techniques is total internal reflection fluorescence (TIRF) microscopy, which allows the visualisation of molecules on the cell surface with the help of fluorescently labelled molecules. In bacteria, it has been used mainly to visualise and follow cytoskeletal proteins MreB and FtsZ^81–83^ and proteins that are involved in cell wall growth because of its localisation near the cell surface. Additionally, a great number of powerful imaging tools have been invented for automated high-throughput quantification of both fluorescence and cell shape microscopy images^84–87^.

Nowadays, a wide range of super-resolution microscopy techniques, such as photoactivated localisation microscopy (PALM)^88^, fluorescence PALM^89^ and stochastic reconstruction microscopy (STORM)^90^, are available. These techniques are based on the ability of fluorescent probes to be activated and re-activated after photobleaching relying on photoactivatable and photoswitchable fluorescent dyes and proteins^91^. Although it has not yet been extensively applied in the field, this technique is becoming more popular and has been used to study cell wall synthesis in *E. coli*^92^** or to visualise modified NAM to track PG dynamics^43^.

Single-molecule imaging revolutionised our understanding of protein localisation and kinetics and represents a useful addition to biochemical studies of protein interactions. Combining these techniques with single-particle tracking is a useful tool that makes it possible to quantify molecular motion inside living cells.

## Conclusions

After decades of studying the composition of PG, great progress has been made in understanding PG dynamics and biosynthesis, aided by the technical advances presented in this review. In PG chemistry, coupling high-throughput analysis with chemometrics allowed us to process large data sets and get an overview of the variability in different species and the chemical composition. The development in microscopy techniques and tools, such as FDAAs, helped us to visualise various events. Structural studies benefited mainly from progress in cryo-EM, which enables us to study the 3D structure of the specimen and proved to be useful in cases when protein crystallisation presents a bottleneck. In the future, the field would greatly benefit from improved imaging strategies such as high-throughput microscopy screens, which would considerably reduce imaging times and increase the number of conditions, as well as from the development of better fluorophores and labelling approaches. Coupling these methods with computational modelling will allow us to gain a broader understanding of cell wall biogenesis.

## References

[1] Vollmer W Blanot D de Pedro MA: Peptidoglycan structure and architecture. *FEMS Microbiol Rev.* 2008; 32(2): 149–67. 10.1111/j.1574-6976.2007.00094.x 18194336

[2] Barreteau H Kovac A Boniface A: Cytoplasmic steps of peptidoglycan biosynthesis. *FEMS Microbiol Rev.* 2008; 32(2): 168–207. 10.1111/j.1574-6976.2008.00104.x 18266853

[3] Bouhss A Trunkfield AE Bugg TDH: The biosynthesis of peptidoglycan lipid-linked intermediates. *FEMS Microbiol Rev.* 2008; 32(2): 208–33. 10.1111/j.1574-6976.2007.00089.x 18081839

[4] Thanbichler M: Synchronization of chromosome dynamics and cell division in bacteria. *Cold Spring Harb Perspect Biol.* 2010; 2(1): a000331. 10.1101/cshperspect.a000331 20182599 PMC2827906

[5] Meeske AJ Riley EP Robins WP: SEDS proteins are a widespread family of bacterial cell wall polymerases. *Nature.* 2016; 537(7622): 634–8. 10.1038/nature19331 27525505 PMC5161649

[6] Yadav AK Espaillat A Cava F: Bacterial Strategies to Preserve Cell Wall Integrity Against Environmental Threats. *Front Microbiol.* 2018; 9: 2064. 10.3389/fmicb.2018.02064 30233540 PMC6127315

[7] Zhao H Patel V Helmann JD: Don't let sleeping dogmas lie: New views of peptidoglycan synthesis and its regulation. *Mol Microbiol.* 2017; 106(6): 847–60. 10.1111/mmi.13853 28975672 PMC5720918

[8] Park JT: Uridine-5'-pyrophosphate derivatives. II. Isolation from *Staphylococcus aureus*. *J Biol Chem.* 1952; 194(2): 877–84. 14927682

[9] Mitchell P Moyle J: The glycerol-phospho-protein complex envelope of *Micrococcus pyogenes*. *J Gen Microbiol.* 1951; 5(5 Suppl): 981–92. 10.1099/00221287-5-5-981 14908035

[10] Salton MRJ: The nature of the cell walls of some gram-positive and gram-negative bacteria. *Biochim Biophys Acta.* 1952; 9(3): 334–5. 10.1016/0006-3002(52)90172-8 12997500

[11] Park JT Strominger JL: Mode of action of penicillin. *Science.* 1957; 125(3238): 99–101. 10.1126/science.125.3238.99 13390969

[12] Weidel W Frank H Martin HH: The rigid layer of the cell wall of *Escherichia coli* strain B. *J Gen Microbiol.* 1960; 22: 158–66. 10.1099/00221287-22-1-158 13843470

[13] Schleifer KH Kandler O: Peptidoglycan types of bacterial cell walls and their taxonomic implications. *Bacteriol Rev.* 1972; 36(4): 407–77. 4568761 10.1128/br.36.4.407-477.1972 PMC408328

[14] Glauner B Höltje JV Schwarz U: The composition of the murein of *Escherichia coli*. *J Biol Chem.* 1988; 263(21): 10088–95. 10.1016/S0021-9258(19)81481-3 3292521

[15] Markiewicz Z Glauner B Schwarz U: Murein structure and lack of DD- and LD-carboxypeptidase activities in Caulobacter crescentus. *J Bacteriol.* 1983; 156(2): 649–55. 10.1128/JB.156.2.649-655.1983 6630150 PMC217879

[16] Desmarais SM de Pedro MA Cava F: Peptidoglycan at its peaks: How chromatographic analyses can reveal bacterial cell wall structure and assembly. *Mol Microbiol.* 2013; 89(1): 1–13. 10.1111/mmi.12266 23679048 PMC3694805

[17] van Teeseling MCF Mesman RJ Kuru E: Anammox Planctomycetes have a peptidoglycan cell wall. *Nat Commun.* 2015; 6: 6878. 10.1038/ncomms7878 25962786 PMC4432595

[18] Kühner D Stahl M Demircioglu DD: From cells to muropeptide structures in 24 h: Peptidoglycan mapping by UPLC-MS. *Sci Rep.* 2014; 4: 7494. 10.1038/srep07494 25510564 PMC4267204

[19] Alvarez L Hernandez SB de Pedro MA: Ultra-Sensitive, High-Resolution Liquid Chromatography Methods for the High-Throughput Quantitative Analysis of Bacterial Cell Wall Chemistry and Structure. *Methods Mol Biol.* 2016; 1440: 11–27. 10.1007/978-1-4939-3676-2_2 27311661

[20] Espaillat A Forsmo O El Biari K: Chemometric Analysis of Bacterial Peptidoglycan Reveals Atypical Modifications That Empower the Cell Wall against Predatory Enzymes and Fly Innate Immunity. *J Am Chem Soc.* 2016; 138(29): 9193–204. 10.1021/jacs.6b04430 27337563

[21] Kumar K Cava F: Principal coordinate analysis assisted chromatographic analysis of bacterial cell wall collection: A robust classification approach. *Anal Biochem.* 2018; 550: 8–14. 10.1016/j.ab.2018.04.008 29649471

[22] Wientjes FB Woldringh CL Nanninga N: Amount of peptidoglycan in cell walls of gram-negative bacteria. *J Bacteriol.* 1991; 173(23): 7684–91. 10.1128/jb.173.23.7684-7691.1991 1938964 PMC212537

[23] Irazoki O Hernandez SB Cava F: Peptidoglycan Muropeptides: Release, Perception, and Functions as Signaling Molecules. *Front Microbiol.* 2019; 10: 500. 10.3389/fmicb.2019.00500 30984120 PMC6448482

[24] Tosoni G Conti M Diaz Heijtz R: Bacterial peptidoglycans as novel signaling molecules from microbiota to brain. *Curr Opin Pharmacol.* 2019; 48: 107–13. 10.1016/j.coph.2019.08.003 31557694

[25] Boudreau MA Fisher JF Mobashery S: Messenger functions of the bacterial cell wall-derived muropeptides. *Biochemistry.* 2012; 51(14): 2974–90. 10.1021/bi300174x 22409164 PMC3345243

[26] Dworkin J: The medium is the message: Interspecies and interkingdom signaling by peptidoglycan and related bacterial glycans. *Annu Rev Microbiol.* 2014; 68: 137–54. 10.1146/annurev-micro-091213-112844 24847956

[27] Gust AA: Peptidoglycan Perception in Plants. *PLoS Pathog.* 2015; 11(12): e1005275. 10.1371/journal.ppat.1005275 26679352 PMC4683077

[28] Meroueh SO Bencze KZ Hesek D: Three-dimensional structure of the bacterial cell wall peptidoglycan. *Proc Natl Acad Sci U S A.* 2006; 103(2): 4404–9. 10.1073/pnas.0510182103 16537437 PMC1450184

[29] Sharif S Singh M Kim SJ: *Staphylococcus aureus* peptidoglycan tertiary structure from carbon-13 spin diffusion. *J Am Chem Soc.* 2009; 131(20): 7023–30. 10.1021/ja808971c 19419167 PMC2778264

[30] Kim SJ Singh M Preobrazhenskaya M: *Staphylococcus aureus* peptidoglycan stem packing by rotational-echo double resonance NMR spectroscopy. *Biochemistry.* 2013; 52(21): 3651–9. 10.1021/bi4005039 23617832 PMC3796188

[31] Kim SJ Chang J Singh M: Peptidoglycan architecture of Gram-positive bacteria by solid-state NMR. *Biochim Biophys Acta.* 2015; 1848(1 Pt B): 350–62. 10.1016/j.bbamem.2014.05.031 24915020 PMC4258515

[32] Yang H Singh M Kim SJ: Characterization of the tertiary structure of the peptidoglycan of Enterococcus faecalis. *Biochim Biophys Acta Biomembr.* 2017; 1859(11): 2171–80. 10.1016/j.bbamem.2017.08.003 28784459 PMC5610627

[33] Tiyanont K Doan T Lazarus MB: Imaging peptidoglycan biosynthesis in Bacillus subtilis with fluorescent antibiotics. *Proc Natl Acad Sci U S A.* 2006; 103(29): 11033–8. 10.1073/pnas.0600829103 16832063 PMC1544169

[34] Sizemore RK Caldwell JJ Kendrick AS: Alternate gram staining technique using a fluorescent lectin. *Appl Environ Microbiol.* 1990; 56(7): 2245–7. 10.1128/AEM.56.7.2245-2247.1990 1697149 PMC184591

[35] de Pedro MA Quintela JC Höltje JV: Murein segregation in Escherichia coli. *J Bacteriol.* 1997; 179(9): 2823–34. 10.1128/jb.179.9.2823-2834.1997 9139895 PMC179041

[36] Cava F de Pedro MA Lam H: Distinct pathways for modification of the bacterial cell wall by non-canonical D-amino acids. *EMBO J.* 2011; 30(16): 3442–53. 10.1038/emboj.2011.246 21792174 PMC3160665

[37] Bugg TD Dutka-Malen S Arthur M: Identification of vancomycin resistance protein VanA as a D-alanine:D-alanine ligase of altered substrate specificity. *Biochemistry.* 1991; 30(8): 2017–21. 10.1021/bi00222a002 1998664

[38] Kuru E Hughes HV Brown PJ: In Situ probing of newly synthesized peptidoglycan in live bacteria with fluorescent D-amino acids. *Angew Chem Int Ed Engl.* 2012; 51(50): 12519–23. 10.1002/anie.201206749 23055266 PMC3589519

[39] Hsu YP Booher G Egan A: d-Amino Acid Derivatives as in Situ Probes for Visualizing Bacterial Peptidoglycan Biosynthesis. *Acc Chem Res.* 2019; 52(9): 2713–22. 10.1021/acs.accounts.9b00311 31419110

[40] Liechti GW Kuru E Hall E: A new metabolic cell-wall labelling method reveals peptidoglycan in *Chlamydia trachomatis*. *Nature.* 2014; 506(7489): 507–10. 10.1038/nature12892 24336210 PMC3997218

[41] Siegrist MS Whiteside S Jewett JC: (D)-Amino acid chemical reporters reveal peptidoglycan dynamics of an intracellular pathogen. *ACS Chem Biol.* 2013; 8(3): 500–5. 10.1021/cb3004995 23240806 PMC3601600

[42] Shieh P Siegrist MS Cullen AJ: Imaging bacterial peptidoglycan with near-infrared fluorogenic azide probes. *Proc Natl Acad Sci U S A.* 2014; 111(15): 5456–61. 10.1073/pnas.1322727111 24706769 PMC3992625

[43] Liang H DeMeester KE Hou CW: Metabolic labelling of the carbohydrate core in bacterial peptidoglycan and its applications. *Nat Commun.* 2017; 8: 15015. 10.1038/ncomms15015 28425464 PMC5411481

[44] Hsu YP Hall E Booher G: Fluorogenic d-amino acids enable real-time monitoring of peptidoglycan biosynthesis and high-throughput transpeptidation assays. *Nat Chem.* 2019; 11(4): 335–341. 10.1038/s41557-019-0217-x 30804500 PMC6444347

[45] Chao Y Zhang T: Optimization of fixation methods for observation of bacterial cell morphology and surface ultrastructures by atomic force microscopy. *Appl Microbiol Biotechnol.* 2011; 92(2): 381–92. 10.1007/s00253-011-3551-5 21881891 PMC3181414

[46] Formanek H Formanek S: Specific staining for electron microscopy of murein sacculi of bacterial cell walls. *Eur J Biochem.* 1970; 17(1): 78–84. 10.1111/j.1432-1033.1970.tb01137.x 4098764

[47] Alsteens D Verbelen C Dague E: Organization of the mycobacterial cell wall: A nanoscale view. *Pflugers Arch.* 2008; 456(1): 117–25. 10.1007/s00424-007-0386-0 18043940

[48] Dupres V Alsteens D Pauwels K: *In vivo* imaging of S-layer nanoarrays on *Corynebacterium glutamicum*. *Langmuir.* 2009; 25(17): 9653–5. 10.1021/la902238q 19642621

[49] Turner RD Mesnage S Hobbs JK: Molecular imaging of glycan chains couples cell-wall polysaccharide architecture to bacterial cell morphology. *Nat Commun.* 2018; 9(1): 1263. 10.1038/s41467-018-03551-y 29593214 PMC5871751

[50] Matias VRF Beveridge TJ: Cryo-electron microscopy reveals native polymeric cell wall structure in *Bacillus subtilis* 168 and the existence of a periplasmic space. *Mol Microbiol.* 2005; 56(1): 240–51. 10.1111/j.1365-2958.2005.04535.x 15773993

[51] Matias VRF Al-Amoudi A Dubochet J: Cryo-transmission electron microscopy of frozen-hydrated sections of *Escherichia coli* and *Pseudomonas aeruginosa*. *J Bacteriol.* 2003; 185(20): 6112–8. 10.1128/jb.185.20.6112-6118.2003 14526023 PMC225031

[52] McIntosh R Nicastro D Mastronarde D: New views of cells in 3D: An introduction to electron tomography. *Trends Cell Biol.* 2005; 15(1): 43–51. 10.1016/j.tcb.2004.11.009 15653077

[53] Pilhofer M Aistleitner K Biboy J: Discovery of chlamydial peptidoglycan reveals bacteria with murein sacculi but without FtsZ. *Nat Commun.* 2013; 4: 2856. 10.1038/ncomms3856 24292151 PMC3847603

[54] Zuber B Chami M Houssin C: Direct visualization of the outer membrane of mycobacteria and corynebacteria in their native state. *J Bacteriol.* 2008; 190(16): 5672–80. 10.1128/JB.01919-07 18567661 PMC2519390

[55] Hoffmann C Leis A Niederweis M: Disclosure of the mycobacterial outer membrane: Cryo-electron tomography and vitreous sections reveal the lipid bilayer structure. *Proc Natl Acad Sci U S A.* 2008; 105(10): 3963–7. 10.1073/pnas.0709530105 18316738 PMC2268800

[56] Gan L Chen S Jensen GJ: Molecular organization of Gram-negative peptidoglycan. *Proc Natl Acad Sci U S A.* 2008; 105(48): 18953–7. 10.1073/pnas.0808035105 19033194 PMC2596242

[57] Vollmer W Seligman SJ: Architecture of peptidoglycan: More data and more models. *Trends Microbiol.* 2010; 18(2): 59–66. 10.1016/j.tim.2009.12.004 20060721

[58] Hayhurst EJ Kailas L Hobbs JK: Cell wall peptidoglycan architecture in *Bacillus subtilis*. *Proc Natl Acad Sci U S A.* 2008; 105(38): 14603–8. 10.1073/pnas.0804138105 18784364 PMC2567149

[59] Beeby M Gumbart JC Roux B: Architecture and assembly of the Gram-positive cell wall. *Mol Microbiol.* 2013; 88(4): 664–72. 10.1111/mmi.12203 23600697 PMC3663049

[60] Li K Yuan XX Sun HM: Atomic Force Microscopy of Side Wall and Septa Peptidoglycan From *Bacillus subtilis* Reveals an Architectural Remodeling During Growth. *Front Microbiol.* 2018; 9: 620. 10.3389/fmicb.2018.00620 29651285 PMC5884923

[61] Pasquina-Lemonche L Burns J Turner RD: The architecture of the Gram-positive bacterial cell wall. *Nature.* 2020; 582(7811): 294–7. 10.1038/s41586-020-2236-6 32523118 PMC7308169

[62] Gumbart JC Beeby M Jensen GJ: *Escherichia coli* peptidoglycan structure and mechanics as predicted by atomic-scale simulations. *PLoS Comput Biol.* 2014; 10(2): e1003475. 10.1371/journal.pcbi.1003475 24586129 PMC3930494

[63] Goodsell DS Franzen MA Herman T: From Atoms to Cells: Using Mesoscale Landscapes to Construct Visual Narratives. *J Mol Biol.* 2018; 430(21): 3954–68. 10.1016/j.jmb.2018.06.009 29885327 PMC6186495

[64] Nguyen LT Gumbart JC Beeby M: Coarse-grained simulations of bacterial cell wall growth reveal that local coordination alone can be sufficient to maintain rod shape. *Proc Natl Acad Sci U S A.* 2015; 112(28): E3689–98. 10.1073/pnas.1504281112 26130803 PMC4507204

[65] Nguyen LT Oikonomou CM Ding HJ: Simulations suggest a constrictive force is required for Gram-negative bacterial cell division. *Nat Commun.* 2019; 10(1): 1259. 10.1038/s41467-019-09264-0 30890709 PMC6425016

[66] Young KD: The selective value of bacterial shape. *Microbiol Mol Biol Rev.* 2006; 70(3): 660–703. 10.1128/MMBR.00001-06 16959965 PMC1594593

[67] Dalia AB Weiser JN: Minimization of bacterial size allows for complement evasion and is overcome by the agglutinating effect of antibody. *Cell Host Microbe.* 2011; 10(5): 486–96. 10.1016/j.chom.2011.09.009 22100164 PMC3222866

[68] Weiser JN: The battle with the host over microbial size. *Curr Opin Microbiol.* 2013; 16(1): 59–62. 10.1016/j.mib.2013.01.001 23395472 PMC3622179

[69] Shiomi D Sakai M Niki H: Determination of bacterial rod shape by a novel cytoskeletal membrane protein. *EMBO J.* 2008; 27(23): 3081–91. 10.1038/emboj.2008.234 19008860 PMC2599877

[70] Campos M Govers SK Irnov I: Genomewide phenotypic analysis of growth, cell morphogenesis, and cell cycle events in *Escherichia coli*. *Mol Syst Biol.* 2018; 14(6): e7573. 10.15252/msb.20177573 29941428 PMC6018989

[71] Peters JM Colavin A Shi H: A Comprehensive, CRISPR-based Functional Analysis of Essential Genes in Bacteria. *Cell.* 2016; 165(6): 1493–506. 10.1016/j.cell.2016.05.003 27238023 PMC4894308

[72] French S Côté JP Stokes JM: Bacteria Getting into Shape: Genetic Determinants of *E. coli* Morphology. *MBio.* 2017; 8(2): e01977–16. 10.1128/mBio.01977-16 28270582 PMC5340871

[73] Zahir T Camacho R Vitale R: High-throughput time-resolved morphology screening in bacteria reveals phenotypic responses to antibiotics. *Commun Biol.* 2019; 2: 269 10.1038/s42003-019-0480-9 31341968 PMC6650389

[74] Huang KC Mukhopadhyay R Wen B: Cell shape and cell-wall organization in Gram-negative bacteria. *Proc Natl Acad Sci U S A.* 2008; 105(49): 19282–7. 10.1073/pnas.0805309105 19050072 PMC2592989

[75] Si F Li D Cox SE: Invariance of Initiation Mass and Predictability of Cell Size in *Escherichia coli*. *Curr Biol.* 2017; 27(9): 1278–87. 10.1016/j.cub.2017.03.022 28416114 PMC5474944

[76] Wang P Robert L Pelletier J: Robust growth of *Escherichia coli*. *Curr Biol.* 2010; 20(12): 1099–103. 10.1016/j.cub.2010.04.045 20537537 PMC2902570

[77] Sauls JT Cox SE Do Q: Control of Bacillus subtilis Replication Initiation during Physiological Transitions and Perturbations. *mBio.* 2019; 10(6): e02205–19. 10.1128/mBio.02205-19 31848269 PMC6918070

[78] Sauls JT Schroeder JW Brown SD: Mother machine image analysis with MM3. *bioRxiv.* 2019; 4–7. 10.1101/810036

[79] Lugagne JB Lin H Dunlop MJ: DeLTA: Automated cell segmentation, tracking, and lineage reconstruction using deep learning. *PLoS Comput Biol.* 2020; 16(4): e1007673. 10.1371/journal.pcbi.1007673 32282792 PMC7153852

[80] Kühn J Briegel A Mörschel E: Bactofilins, a ubiquitous class of cytoskeletal proteins mediating polar localization of a cell wall synthase in Caulobacter crescentus. *EMBO J.* 2010; 29(2): 327–39. 10.1038/emboj.2009.358 19959992 PMC2824468

[81] Hussain S Wivagg CN Szwedziak P: MreB filaments align along greatest principal membrane curvature to orient cell wall synthesis. *eLife.* 2018; 7: e32471. 10.7554/eLife.32471 29469806 PMC5854468

[82] Bisson-Filho AW Hsu YP Squyres GR: Treadmilling by FtsZ filaments drives peptidoglycan synthesis and bacterial cell division. *Science.* 2017; 355(6326): 739–43. 10.1126/science.aak9973 28209898 PMC5485650

[83] Billaudeau C Yao Z Cornilleau C: MreB Forms Subdiffraction Nanofilaments during Active Growth in *Bacillus subtilis*. *mBio.* 2019; 10(1): e01879-18. 10.1128/mBio.01879-18 30696741 PMC6355991

[84] Ducret A Quardokus EM Brun YV: MicrobeJ, a tool for high throughput bacterial cell detection and quantitative analysis. *Nat Microbiol.* 2016; 1(7): 16077. 10.1038/nmicrobiol.2016.77 27572972 PMC5010025

[85] Paintdakhi A Parry B Campos M: Oufti: An integrated software package for high-accuracy, high-throughput quantitative microscopy analysis. *Mol Microbiol.* 2016; 99(4): 767–77. 10.1111/mmi.13264 26538279 PMC4752901

[86] Stylianidou S Brennan C Nissen SB: SuperSegger: Robust image segmentation, analysis and lineage tracking of bacterial cells. *Mol Microbiol.* 2016; 102(4): 690–700. 10.1111/mmi.13486 27569113

[87] Ursell T Lee TK Shiomi D: Rapid, precise quantification of bacterial cellular dimensions across a genomic-scale knockout library. *BMC Biol.* 2017; 15(1): 17. 10.1186/s12915-017-0348-8 28222723 PMC5320674

[88] Betzig E Patterson GH Sougrat R: Imaging intracellular fluorescent proteins at nanometer resolution. *Science.* 2006; 313(5793): 1642–5. 10.1126/science.1127344 16902090

[89] Hess ST Girirajan TPK Mason MD: Ultra-high resolution imaging by fluorescence photoactivation localization microscopy. *Biophys J.* 2006; 91(11): 4258–72. 10.1529/biophysj.106.091116 16980368 PMC1635685

[90] Rust MJ Bates M Zhuang X: Sub-diffraction-limit imaging by stochastic optical reconstruction microscopy (STORM). * Nat Methods.* 2006; 3(10): 793–5. 10.1038/nmeth929 16896339 PMC2700296

[91] Fernández-Suárez M Ting AY: Fluorescent probes for super-resolution imaging in living cells. *Nat Rev Mol Cell Biol.* 2008; 9(12): 929–43. 10.1038/nrm2531 19002208

[92] Turner RD Hurd AF Cadby A: Cell wall elongation mode in Gram-negative bacteria is determined by peptidoglycan architecture. *Nat Commun.* 2013; 4: 1496. 10.1038/ncomms2503 23422664 PMC3586723

